# Validation of Non-Restrictive Inertial Gait Analysis of Individuals with Incomplete Spinal Cord Injury in Clinical Settings

**DOI:** 10.3390/s22114237

**Published:** 2022-06-02

**Authors:** Roushanak Haji Hassani, Romina Willi, Georg Rauter, Marc Bolliger, Thomas Seel

**Affiliations:** 1 Bio-Inspired RObots for MEDicine-Laboratory (BIROMED-Lab), Deptartment of Biomedical Engineering, University of Basel, 4001 Basel, Switzerland; roushanak.hajihassani@unibas.ch (R.H.H.); georg.rauter@unibas.ch (G.R.); 2 Spinal Cord Injury Center, Balgrist University Hospital, University of Zurich, 8008 Zurich, Switzerland; romina.willi@balgrist.ch (R.W.); marc.bolliger@balgrist.ch (M.B.); 3 Department Artificial Intelligence in Biomedical Engineering, Friedrich-Alexander-Universität Erlangen-Nürnberg, 91054 Erlangen, Germany

**Keywords:** inertial measurement units, optical motion capture, range of motion, temporal gait parameters, inertial gait analysis, incomplete spinal cord injury, validation, clinical gait assessment

## Abstract

Inertial Measurement Units (IMUs) have gained popularity in gait analysis and human motion tracking, and they provide certain advantages over stationary line-of-sight-dependent Optical Motion Capture (OMC) systems. IMUs appear as an appropriate alternative solution to reduce dependency on bulky, room-based hardware and facilitate the analysis of walking patterns in clinical settings and daily life activities. However, most inertial gait analysis methods are unpractical in clinical settings due to the necessity of precise sensor placement, the need for well-performed calibration movements and poses, and due to distorted magnetometer data in indoor environments as well as nearby ferromagnetic material and electronic devices. To address these limitations, recent literature has proposed methods for self-calibrating magnetometer-free inertial motion tracking, and acceptable performance has been achieved in mechanical joints and in individuals without neurological disorders. However, the performance of such methods has not been validated in clinical settings for individuals with neurological disorders, specifically individuals with incomplete Spinal Cord Injury (iSCI). In the present study, we used recently proposed inertial motion-tracking methods, which avoid magnetometer data and leverage kinematic constraints for anatomical calibration. We used these methods to determine the range of motion of the Flexion/Extension (F/E) hip and Abduction/Adduction (A/A) angles, the F/E knee angles, and the Dorsi/Plantar (D/P) flexion ankle joint angles during walking. Data (IMU and OMC) of five individuals with no neurological disorders (control group) and five participants with iSCI walking for two minutes on a treadmill in a self-paced mode were analyzed. For validation purposes, the OMC system was considered as a reference. The mean absolute difference (MAD) between calculated range of motion of joint angles was 5.00°, 5.02°, 5.26°, and 3.72° for hip F/E, hip A/A, knee F/E, and ankle D/P flexion angles, respectively. In addition, relative stance, swing, double support phases, and cadence were calculated and validated. The MAD for the relative gait phases (stance, swing, and double support) was 1.7%, and the average cadence error was 0.09 steps/min. The MAD values for RoM and relative gait phases can be considered as clinically acceptable. Therefore, we conclude that the proposed methodology is promising, enabling non-restrictive inertial gait analysis in clinical settings.

## 1. Introduction

In pre-clinical Spinal Cord Injury (SCI) research, there has been a steady increase in the number of experimental therapeutics and active rehabilitation training paradigms that aim at improving musculoskeletal outcomes. Some of these very promising interventions are currently being investigated in clinical trials. If these interventions show beneficial effects for individuals with SCI, there is a need to understand the mechanisms behind the observed improvements. However, the established clinical measures for lower-limb function are subjective and qualitative [1], which makes it challenging to identify whether performance improvements have been achieved by recovery or compensatory mechanisms [2].

Improvement mechanisms can be identified by quantifying gait performance via biomechanical analysis (kinematics and kinetics). Such assessments are usually carried out through optical motion tracking systems (OMC)s in a controlled lab environment, and are considered the gold standard due to sufficient accuracy [3]. However, OMCs require expensive equipment, ample lab space, and highly trained examiners to correctly place the reflective markers. Furthermore, long preparation times (e.g., for marker placement and calibration poses) are needed prior to each trial, and post-processing of the data requires likewise large amounts of time and expert knowledge (e.g., for labeling and reconstructing data of missing markers due to occlusion) [4]. On the other hand, wearable systems for “Inertial Motion Capture (IMC)” are gaining popularity among researchers as a promising alternative tool for less-restrictive motion tracking indoors and outdoors [5]. IMC systems include one or several lightweight Inertial Measurement Units (IMU)s, which typically consist of a tri-axial accelerometer, a tri-axial gyroscope, and a tri-axial magnetometer that measure accelerations, angular rates, and magnetic field vectors, respectively, in intrinsic coordinate systems. IMUs can be used as an assessment tool in daily life activities due to portability for optimization of treatment, individualization of the rehabilitation process, and quantification of the recovery state of individuals with neurological disorders [6,7,8]. However, the desired information, such as the Range of Motion (RoM) of joint angles and temporal gait characteristics, needs to be extracted with robust methods, which can be applied to patient data and used in clinical settings rather than only in ideal laboratory environments.

Several methodologies have been proposed and validated for inertial gait analysis; however, these methodologies are restrictive when it comes to being used in clinical settings with patients. The methods either depend on optical markers combined with IMUs [9] or require precise IMU mounting [10] and predefined sequences of particular postures or movements [11,12,13,14,15] to identify sensor-to-body alignments before dynamic trials. Even though precise joint angle estimation is achieved by employing additional hardware or predefined postures/movements, such approaches are restrictive and error-prone in individuals with neurological pathologies who (i) suffer from movement disorders, (ii) cannot perform predefined calibration movements with sufficient precision, or (iii) cannot follow the predefined pace of tasks. This leads to failure of the alignment process and results in an inaccurate joint angle estimation. Furthermore, the magnetic field in indoor environments is disturbed due to soft/hard iron effects or nearby ferromagnetic material and electronic devices [16], which results in incorrect heading angles and severe drift effects.

To overcome the mentioned limitations, less-restrictive and calibration-free algorithms have been proposed recently for finding automatic sensor alignment [17], correcting relative heading offset [18], and determining spatiotemporal gait parameters [19]. The proposed methods have been tested and validated using 3D printed mechanical joints [17,18] and healthy participants. However, their performance was not evaluated and validated in individuals with neurological disorders such as people with incomplete SCI (iSCI). Thus, the main focus of this paper is to address this shortcoming by:

- Combining less-restrictive approaches [17,18,19] for plug-and-play inertial gait analysis;
- Validating the temporal gait parameters and the estimated RoM of hip, knee, and ankle with an OMC reference system using the plug-in gait model [20].

## 2. Materials and Methods

### 2.1. Measurement System

InvenSense MEMS sensors with integrated IMUs (MPU9250) from Noraxon (Ultium EMG, Noraxon, Scottsdale, AZ, USA) were used to measure the angular velocity (±2000 degrees/s), acceleration (±16 g), and magnetic field amplitude (±4800 µT). The sampling rate was set to 400Hz. An OMC system comprising 27 infrared cameras set to a sampling rate of 200Hz (Vicon, Bilston, UK) was used as a reference tool. A total of 42 retro-reflective spherical markers (14mm) were placed on bony landmarks according to the plug-in gait model [20].

### 2.2. Participants

Five control participants (3 females; age: 31.6(±4.6) years; height: 180.2(±16.3) cm; weight: 66.2(±8.7) kg) and five participants with iSCI (2 females; age: 45(±17.2) years; height: 173.2(±5.40) cm; weight: 79.6(±21.6) kg) were recruited for the study. The inclusion criteria for iSCI participants were age ≥ 18 years and acute or chronic SCI. The exclusion criteria were orthopaedic problems, major psychosis or depression, and a history of severe heart conditions. The anthropomorphic data of all participants and lesion level, impairment scale, and Walking Index for Spinal Cord Injury II (WISCI II) of the participants with iSCI are presented in Table 1. Ethical approval was obtained for this study from the local ethics committee of the Canton of Zurich, Zurich, Switzerland (BASEC-Nr. 2020-01473) and conducted following the Declaration of Helsinki.

### 2.3. Data Collection

#### 2.3.1. Preparation Procedure—OMC Setup

The cameras were turned on before participant arrival (recommended warm-up duration: 30–90 min before calibration [21]) and were re-calibrated a few minutes before the beginning of the experiment. Participants were equipped with 42 reflective markers that were positioned over bony landmarks using the plug-in gait model (time for preparation and marker placements: 20 min). A static posture, standing without motion for 15 s, head facing straight, legs placed apart, and hands stretched out in a T-position, was recorded for the OMC calibration purpose before the walking trial.

#### 2.3.2. Preparation Procedure—IMC Setup

**Figure 1:**
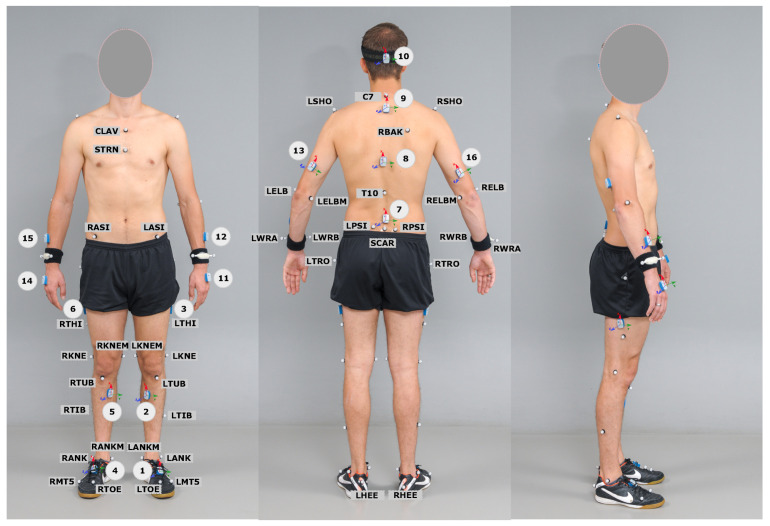
Placement of IMUs and marker placement of the plug-in gait model. A total of 42 reflective markers are placed on bony landmarks of participants, and 16 IMUs are placed based on proposed instructions of the manufacturer. In this study, IMUs 1–7 were used for analysis.

IMUs were placed directly on the body segments (i.e., attached to the skin) based on the proposed manufacturer instructions and secured with medical tapes to avoid wobbling and unwanted movements (time for sensor placement: 7 min). IMU placement was performed as and described in Figure 1 and Table 2.

After positioning all the markers and IMUs, a static posture was recorded for the OMC system. Thereafter, the participants performed two minutes of walking at their comfortable self-selected speed on the treadmill with the activated self-paced mode. Data were collected synchronously from IMUs and reflective markers during the walking trial for further evaluation.

### 2.4. Data Processing and Analysis

#### 2.4.1. Extraction of Temporal and Kinematic Gait Characteristics—OMC System

Gait parameters and kinematic characteristics were extracted by following the standard and well-defined procedures. The raw data collected from the cameras were used to reconstruct each body segment’s motion using the plug-in gait model, which has defined guidelines for data collection, processing, and analysis. First, 3D trajectories are reconstructed from 2D data; the marker data were labeled and scaled based on anthropomorphic data. Subsequently, the participants’ bone length, joint location, and marker position were calculated, and the static gait model was executed. The walking trial was labeled automatically by the implemented static gait model from the static trial, and the Woltring filtering routine [22] was applied to all marker trajectories to ensure a smooth result for calculating kinetics. Then, the dynamic plug-in gait model was executed, and joint kinematics and gait events were extracted based on the placed markers on the heels (RHEE, LHEE) and the toes (RTOE, LTOE).

#### 2.4.2. Gait Temporal Characteristics—IMC System

Gait events and phases were identified using the calibration-free gait assessment method introduced by [19]. The technique used angular rates and acceleration signals from two IMUs placed on the shoes and automatically adapted its parameters to the participant’s gait velocity. It has been shown that the method can overcome the difficulty of identifying gait events in stroke patients’ irregular gait patterns. Using the proposed method in [19], gait events—i.e., Initial Contacts (IC) and Final Contacts (FC)—were extracted from IMUs, which were placed on the shoes. The gait cycles began from the IC of one foot and lasted until the consecutive IC of the same foot. Each gait cycle consists of two main phases: the stance phase and the swing phase. The stance phase, known as the period when the foot is in contact with the ground, begins with the IC of the foot and ends with the FC of the same foot (approximately 60% of one gait cycle). The swing phase begins with the FC of one foot and ends with the IC of the same foot; that is, the period when the foot is not in contact with the ground (approximately 40% of the gait cycle). Double support is a period when both feet are in touch with the ground, which occurs at the beginning (Initial double support phase: phase between IC of one foot to FC of contralateral foot) and end (Terminal double support phase: phase between IC of contralateral to the FC) of the stance phase. Temporal gait parameters such as relative swing, stance, and double-support phases, and cadence were calculated based on the identified gait events and phases, as follows:

- Relative swing phase: swing phase time/stride time;
- Relative stance phase: stance phase time/stride time;
- Relative double support phase: double support time/stride time;
- Cadence: number of steps/minute.

#### 2.4.3. Gait Kinematic Characteristics—IMC System

Gait kinematics, e.g., joint angles from IMU data, can be calculated from the orientation of each IMU attached to the two adjacent segments. The orientation of each IMU is obtainable by fusing the data from the three integrated sensors (9D sensor fusion) [23] inside each unit. However, due to an unreliable magnetic field in the indoor environment, a quaternion-based 6D sensor fusion algorithm was used for orientation estimation. The 6D sensor fusion used strap-down integration of the angular rates and geodetic accelerometer-based drift compensation [24]. However, avoiding magnetometer data in orientation estimation leads to sensor drift on the estimated heading angles. An approach that corrects relative heading errors over time for calculating relative joint angle is proposed in [18,25], and was also applied in this study. The method uses the orientation estimates from 6D inertial sensor fusion and exploits approximate kinematic models and constraints of different joint types to estimate the relative heading between neighboring segments and thereby eliminate the relative heading drift of the magnetometer-free orientations.

**Figure 2:**
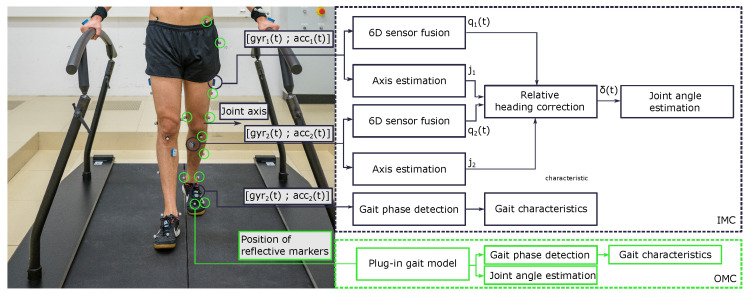
Overview of the data-processing steps in validation of estimated gait kinematics and temporal parameters using IMUs.

Moreover, the relative heading error correction method needs the primary joint axes seen in both adjacent sensor coordinate systems. We estimated joint axes using the approach introduced in [17] in a plug-and-play manner. The plug-and-play method employs gyroscope and accelerometer data of two IMUs attached to the two adjacent segments during walking trials and calculates pairs of dominant joint axes in the sensor coordinate system. There is sign-pairing ambiguity for the particular case of joints whose main axes remain horizontal throughout the motion. The sign-pairing ambiguity was addressed through appropriate sensor attachment, for instance, “the thigh IMU was placed laterally“, “the pelvis IMU was attached roughly upside-up“, and “the shank IMU was placed on anterior and medially along the tibial bone“. Finally, the RoM of the F/E hip angle, F/E knee angle, and D/P flexion ankle angles are determined by the following steps: First, a joint angle α(t) was estimated by combining the less restrictive approaches [17,18,19,20]. Then, the average is removed, and the joint angle data are segmented into stride-wise zero-average joint-angle sequences ${\tilde{\alpha }}_{i}\left(t\right)$, i=1,2,…, using the IMC-based initial contact events. Finally, the stride-wise Range of Motion (RoM) is determined as the difference between the maximum and minimum value of that sequence, as illustrated in Figure 2.

#### 2.4.4. Comparison Matrices

For comparison purposes, we determined the Mean Absolute Difference (MAD) and STandard Deviation of absolute Difference (STDD) of RoM, for each subject over all strides, using OMC and IMC systems as presented in the result section. The OMC was taken as the ground truth. Furthermore, the calculated cadence and relative swing, stance, and double support from the IMC system were compared with the same parameters extracted from the OMC system. The comparison results are presented in the format of (MAD (± STDD)).

## 3. Results

**Figure 3:**
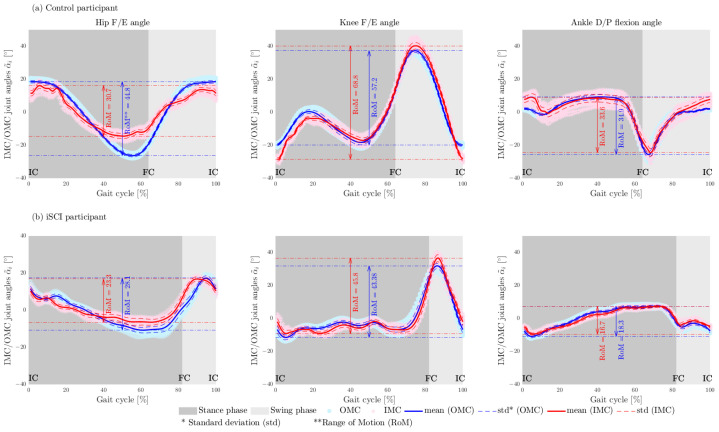
Exemples of RoM of the F/E hip angle, F/E knee angle, and D/P flexion ankle angle for (**a**) one control participant and (**b**) one iSCI participant walking on the treadmill. Red represents the IMU-based joint angles ${\tilde{\alpha }}_{i}\left(t\right)$, and blue represents the corresponding joint angles obtained from OMC system employing the plug-in gait model (used as a reference). The stance and swing phases are shaded by dark and light grey in the background. Initial contact and Final contact are marked as IC and FC in each plot.

All 10 participants’ data obtained from the IMC and OMC were analyzed. In total, 823 strides (control = 450, iSCI = 373) were used in this study to validate the RoM of hip F/E and A/A angles, knee F/E angles, ankle D/P flexion angles, and temporal gait characteristics (relative stance, swing, double support phases, and cadence) estimated with the IMC setup. Data of left knee and hip angles of one control participant and left ankle angle of two participants with iSCI were excluded from analysis due to missing data of an IMU placed on the thigh and an error during post-processing of OMC data, respectively. Hip, knee, and ankle joint angles for one representative control participant and one iSCI participant are presented for both measurement setups in Figure 3. The MAD and STDD of RoM of each joint angle (over each group of participants), in the form of the MAD (± STDD), were calculated and reported in Table 3. In addition, the Root-Mean-Square Error (RMSE) for estimated joint angles in the sagittal plane were 4.75°(±1.15°), 5.36°(±1.4°), and 3.87°(±1.47°).

**Figure 4:**
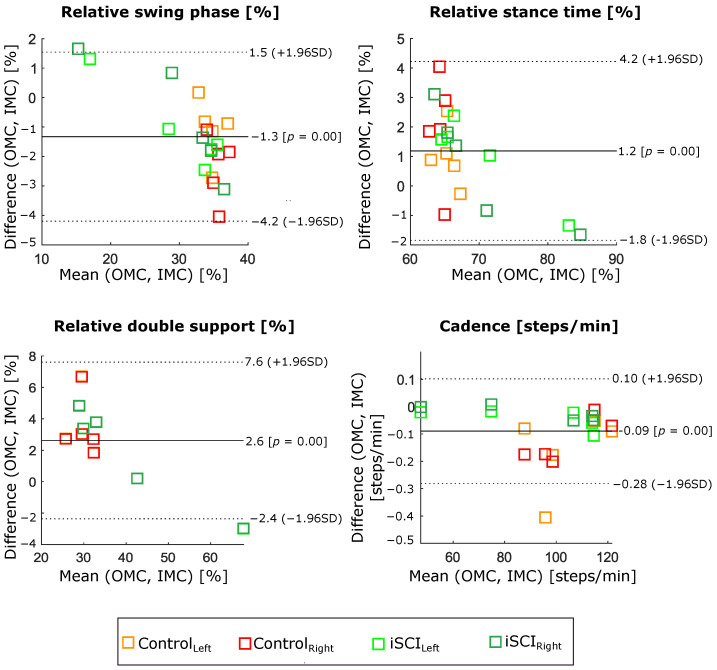
Bland–Altman graphs represent the difference scores of calculated relative swing, stance, double-support phases, and cadence from two IMUs placed on the shoes versus OMC system while the participants (5 controls and 5 iSCIs (823 strides)) were walking on the treadmill.

The IMC system’s estimated gait events and temporal parameters were compared with those characteristics extracted from the OMC system. The agreement of relative stance, swing, and double-support phases, as well as cadence between two measurement systems, is shown in Bland–Altmann diagrams (see Figure 4). Bias and limits of agreement (95% confidence interval of the bias) are represented by solid and dashed lines, respectively. Control and participants with iSCI data are highlighted in different colours. Table 4 shows a detailed comparison of the estimated temporal gait parameters from the IMC system, considering the OMC system as a reference.

## 4. Discussion

This study aimed to validate a combination of recently proposed non-restrictive approaches in inertial and gait analysis [17,18,19,20]. The proposed methods were validated using a 1D mechanical hinge joint and/or control participants. However, the results of a recent study showed that the error of joint-angle estimation on a biological joint is about four times larger than that of a prosthesis, which can be considered a mechanical joint [26]. This current study tested the proposed methods on control and individuals with iSCI and validated the parameters estimated using the IMC system with those obtained from the OMC system. The MAD (±STDD) values of RoM of the estimated angles with respect to the reference system over control and iSCI groups were 5.00°(±2.05°) for hip F/E angles, 5.07°(±1.53°) for hip A/A angles, 5.26°(±1.51°) for knee F/E angles, and 3.72°(±2.08°) for the ankle D/P flexion angles (see Table 3). Figure 3 shows that the difference between the angles estimated by OMC and IMC was larger in ICs and FCs (pre-swing phase) when the legs were accelerated and decelerated, which directly influence the determined range of motion. The results showed that range of motion estimation has a robust performance in both control and participants with iSCI. Furthermore, the obtained accuracy is in the range of clinically acceptable between-system differences, i.e., near or below 5° [27], and it is comparable to the accuracy reported for range-of-motion and joint-angle measurements in the literature [28,29,30]. Therefore, non-restrictive inertial gait analysis methodology can be used in clinical settings.

It is difficult to compare these results with those of other validation studies in the literature because of the differences in the applied procedure including sensor placement, reference system, and applied methods for kinematic and gait parameter calculation. Most of the recently published research recruited healthy participants. However, in clinical settings, the focus is on the investigation of individuals with disturbed daily functional movement. One of the crucial issues with using the state-of-the-art approaches in a clinical setting is the inability of the participants to perform predefined calibration postures and movements that are necessary for the analysis and evaluation of movements using IMUs. It is clear that despite the advancements in inertial gait analysis, there are still some restrictions, such as invalid magnetometer data in the indoor environment and sensor-to-body alignments that render their use in clinical settings inefficient. Therefore, this validation study is the first study to combine and investigate the less restrictive approaches—i.e., orientation estimation without magnetometer data, plug-and-play sensor axes estimation for estimating sensor-to-body alignment, and relative heading error correction methods—on iSCI and control.

However, our findings are comparable with those of approaches that need predefined calibration postures and movements or initialization. Dorschky et al. [28] investigated the accuracy of estimated lower-limb joint angles by exploiting musculoskeletal modelling using OMC as a reference. The Root-Mean-Square Error (RMSE) values of 8.7°, 5.3°, and 4.6° were achieved for the hip, knee, and ankle angles of 10 control participants in the sagittal plane, respectively. Nuesch et al. [30] reported RMSE values of 9.6°, 7.6°, and 4.5° for the hip, knee, and ankle angles, respectively, in the sagittal plane, of over 20 control participants. The sensor-to-body alignment was calculated using 10s recordings of the subject in a predefined, neutral, upright standing pose and performing squatting movements. However, we have achieved a smaller RMSE value (4.75°, 5.36°, and 3.87°) for hip, knee, and ankle joints with a less restrictive approach. Wolfgang et al. reported an RoM error of less than 6.10° for hip and knee angle and an error of 10.6° for ankle angle in the sagittal plane over 28 control participants [31]. The first OMC frame of each walking sequence was used as initialization for the IMU-based kinematics estimation, making the IMU-based analysis highly dependent on the OMC setup. Note that these and the vast majority of all previously proposed methods either rely on predefined calibration poses, which must be performed precisely, or on additional sensor systems, such as OMC. The proposed approach overcomes these limitations and yet consistently achieves smaller errors (5.0°, 5.2°, 3.7°) for the hip, knee and ankle RoM in the sagittal plane.

Furthermore, ICs and FCs of the gait were detected from the data of two IMUs mounted on participants’ shoes to understand joint motions at specific points of gait cycles. Moreover, temporal parameters such as relative gait phases (stance, swing, and double support) and cadence were calculated from the detected gait events and validated against the parameters extracted from the OMC system. The disagreements between OMC and IMC, presented in Table 4, are in good correspondence with previous literature findings. For instance, the MAD concerning the OMC as a reference system was 1.7% for the duration of the gait phases, and 0.09 steps/min for the cadence. These findings are consistent with a recent validation study by Laidig et al. [19], since the same method was applied to our data to extract gait events and parameters. The MAD between 1.14% to 2.26% and 1.16% to 2.22% for the stance and swing phases, respectively, were reported for three (healthy, orthopedic, and neurological) groups of participants. An average error of 0.7 steps/min was reported for the cadence, while our finding was 0.09 steps/min.

Overall, the investigated non-restrictive methods for gait analysis using OMC as a reference system showed acceptable performance. Although the OMC system provides very specialized, detailed gait analysis, particular advantages of the IMC system versus the OMC system made IMC a method of choice for most clinical assessments. Clinically acceptable between-system (IMC, OMC) differences, relatively short preparation time (7 min versus 90 min), lower initial investment and maintenance cost, and infinite measurement space versus one room-bound measurement make the IMC system a proper alternative to OMC in the clinical setting.

However, there are certain limitations in this study that could be addressed in future research. First, the proposed plug-and-play approach facilitates measurements of the RoM of joint angles. It is shown that accurate RoM values are obtained without the need for precise calibration poses. Note that the method could be further extended so that it also provides accurate *absolute* joint-angle measurements. This, however, is outside the scope of the present article. Second, the study used the plug-and-play method with some general assumptions regarding the placement of IMUs in different aspects of the body segments (non-precise location, i.e., in the inner or outer part of the segments) for sign matching. Future investigations can employ automatic sign matching. Third, aside from testing validity, the test–retest reliability of the proposed methods needs to be checked for both groups of individuals. Moreover, investigations are also recommended to validate special gait parameters such as stride width and length. Lastly, inertial gait analysis was performed while participants walked in a the self-paced mode on the treadmill. However, there is a wide range of opinions on whether treadmill walking can replicate overground walking. A study with 17 control participants showed a significant decrease in stance time and increased hip RoM, maximum hip flexion angle, and cadence during treadmill walking [32]. Since the applied non-restrictive algorithms in this study were designed to perform well in different groups of participants (control, iSCI), it is safe to expect that the functionality will not change for hard-floor, overground walking. However, the algorithm should be assessed for uneven surfaces, outdoor surfaces, and walkways with turns.

## 5. Conclusions

This study validated non-restrictive methods that facilitate inertial gait analysis in clinical settings. We focused on validating cadence, gait phases, and range of motion of the joint angles. Anatomical (sensor-to-segment) calibration was achieved by non-restrictive plug-and-play methods that exploit kinematic constraints. This methodology is more practical in clinical settings than previously suggested methods that require precise movements and poses for calibration. Summing up the results, an average Mean Absolute Difference (MAD) of 4.76° was obtained for the range of motion of all joints and both participant groups (five control and five individuals with incomplete spinal cord injury ) using the plug-in gait model of optical motion capture systems as a reference. The MAD, with respect to the reference system, was 1.7% for the gait phase duration and 0.09 steps/min for the cadence. The results confirm that non-restrictive inertial gait analysis systems have the potential to replace stationary gait analysis systems due to ease of use, portability, and accuracy. Future research will focus on the automatic sign matching of the joint axes, measuring the test–retest reliability, and validating additional gait parameters such as stride length and width, and assessing performance on uneven surfaces, outdoors, and walkways with turns.

## Figures and Tables

**Table 1:** Participants’ anthropomorphic data.

Participants	Gender	Age [Year]	Height [cm]	Weight [kg]	Lesion Level (and Impairment Scale)	WISCI II ${}^{a}$
C01	Male	35	173	71	−	−
C02	Female	32	175	60	−	−
C03	Male	37	180	79	−	−
C04	Female	28	165	58	−	−
C05	Female	26	173	63	−	−
P01	Male	65	183	86	C6, ASIA D	20
P02	Male	62	193	100	C7, ASIA D	20
P03	Female	37	167	45	C6, ASIA D	19
P04	Male	28	198	93	L2, ASIA C	20
P05	Female	33	160	74	C5, ASIA D	8
**Mean**(±**std**)	−	38.3 (±13.8)	176.7 (±12.1)	72.9 (±17.1)	−	

^a^ Walking Index for Spinal Cord Injury (WISCI II).

**Table 2:** Description of IMUs placements.

Sensor	Position	Description
1	Left foot	Dorsal side of the left foot
2	Left shank	Anterior and medially along the tibial bone
3	Left thigh	Laterally, 2× palm above knee
4	Right foot	Dorsal side of the right foot
5	Right shank	Anterior and medially along the tibial bone
6	Right thigh	Laterally, 2× palm above knee
7	Pelvis	Body of sacrum

**Table 3:** RoM comparison between IMC and OMC in control and participants with iSCI.

Joints	RoM ${}^{a}$ Comparison [MAD ${}^{b}$ (± STDD ${}^{c}$)]		
	Control (*n* ${}^{d}$ = 5, Strides = 450)	iSCI (*n* = 5, Strides = 373)	Mean (*n* = 10, Strides = 823)
Hip F/E angle [${}^{\circ }$]	5.24(±2.38)	4.76(±1.73)	5.00(±2.05)
Hip A/A angle [${}^{\circ }$]	4.34(±1.77)	5.81(±1.29)	5.07(±1.53)
Knee F/E angle [${}^{\circ }$]	3.34(±1.54)	5.22(±1.48)	**5.26** (± 1.51)
Ankle D/P flexion angle [${}^{\circ }$]	4.29(±2.14)	3.18(±2.02)	3.72(±2.08)
**Mean [${}^{\circ }$]**	4.30(±1.95)	4.74(±1.63)	4.76 (± 1.79)

^a^ Range of Motion (RoM); ^b^ Mean Absolute Difference (MAD); ^c^ STandard Deviation of Difference (STDD); ^d^ Number of participants (n).

**Table 4:** Comparison of gait temporal parameters between IMC and OMC systems in control and participants with iSCI.

Gait Parameters		Comparison of Gait Parameters [MAD ${}^{a}$ (±STDD ${}^{b}$)]	
	Control (*n* = 5, Strides = 450)	iSCI (*n* = 5, Strides = 373)	Mean (*n* = 10, Strides = 823)
Relative swing phase [%]	1.75(±1.23)	1.69(±1.63)	1.72(±1.43)
Relative stance phase [%]	1.71(±1.55)	1.67(±1.62)	1.69(±1.63)
Relative double support [%]	3.40(±1.78)	3.03(±3.01)	1.69(±2.39)
Cadence [steps/min]	0.14(±0.11)	0.04(±0.03)	0.09(±0.07)

^a^ Mean Absolute Difference (MAD); ^b^ STandard Deviation of Difference (STDD).

## Data Availability

Not applicable.

## Abbreviations

The following abbreviations are used in this manuscript:

iSCI	incomplete Spinal Cord Injured
IMU	Inertial Measurement Unit
OMC	Optical Motion Capture
RMSE	Root Mean Square Error
RoME	Range of Motion Error
CSI	Cubic Spline Interpolation
std	standard deviation
MAD	Mean Absolute Difference
IC	Initial Contact
FC	Final Contact
STDD	STandard Deviation of Difference
RoM	Range of Motion
WISCI II	Walking Index for Spinal Cord Injury
F/E	Flexion/Extension
D/P	Dorsi/Plantar
A/A	Abduction/Adduction
RMSE	Root Mean Square Error
